# Post-traumatic elbow stiffness: etiology, risk factors and current treatments

**DOI:** 10.3389/fsurg.2025.1643326

**Published:** 2025-08-26

**Authors:** Maoqiang Fan, Fan Xu, Chen Fei, Yao Liu, Zhongtao Yang, Zhe Song

**Affiliations:** ^1^ Department of Orthopaedic Surgery, Honghui Hospital, Xi'an Jiaotong University, Xi'an, Shaanxi, China; ^2^ Department of Orthopaedic Surgery, Xi'an Medical University, Xi'an, Shaanxi, China

**Keywords:** post-traumatic elbow stiffness, diagnosis, treatment, surgical release, risk factors

## Abstract

Post-traumatic elbow stiffness is a major cause of functional impairment after elbow trauma, affecting 10%–15% of patients. A 50% reduction in elbow range of motion can lead to an 80% decline in upper limb functionality, significantly impairing work performance and social participation. Based on a systematic search of Pub Med, Web of Science, China Knowledge Net and other databases, this paper systematically reviewed the diagnosis and treatment of post-traumatic elbow stiffness. While open and arthroscopic lysis remain effective when nonoperative treatment fails, the timing of surgery and the duration of postoperative immobilization remain controversial. Early and individualized rehabilitation is critical, and future research will focus on optimizing individualized treatment protocols and multidisciplinary treatment modalities.

## 1 Introduction

The elbow joint is a complex structure comprising the humeroulnar, humeroradial, and proximal radioulnar articulations, all encapsulated within a single joint capsule. Structural integrity is provided by the ulnar collateral, radial collateral, and annular ligaments. The typical range of motion encompasses approximately 135°–145°of flexion, −10°−0°of extension, and 80°–90°of pronation and supination (1). Post-traumatic elbow stiffness (PTES) is a prevalent complication following elbow injury, with an occurrence rate of 10%–15% among trauma patients (2). Traditionally, it was believed that 100° arcs of flexion-extension and forearm rotation were sufficient for most daily activities (3). However, recent evidence suggests that the functional range of motion required for common tasks, such as using a mobile phone or typing, may be more extensive (4). Notably, a 50% reduction in elbow mobility can result in an 80% decline in upper limb function, significantly impairing work productivity and social engagement (5). Furthermore, prolonged joint stiffness and limited range of motion may lead to detrimental psychological consequences, including anxiety and depression, adversely affecting mental well-being and imposing a strain on healthcare systems.

## 2 Etiology and risk factors

### 2.1 Etiology

#### 2.1.1 Peripheral soft tissue contracture

Posttraumatic elbow stiffness is often associated with fibrotic contracture of the elbow capsule, characterized by thickening and stiffening of the capsular tissue (6). Histological analysis has revealed increased collagen cross-linking, decreased proteoglycan content, and reduced water content, indicative of extracellular matrix proliferation and the presence of highly contractile myofibroblasts (3). *In vivo* studies have identified the role of transforming growth factor beta (TGF-β) in promoting myofibroblast differentiation, further supporting the involvement of these cells in the development of posttraumatic elbow stiffness (7). Lumican belongs to the class II small leucine-rich proteoglycan superfamily, which constitutes collagen fibrils in the extracellular matrix. A study (8) conducted by Xiao et al. revealed that lumican exhibits a pro-fibrotic function and may promote the occurrence of joint contracture by activating the TGF-β signaling pathway. A recent study (9) employed gene expression profiling to elucidate the molecular mechanisms underlying capsular contracture. Key pivotal genes, such as SPP1, IBSP, MMP13, and MYO1A, were found to play a significant role in the fibrotic process. Additionally, the study identified hsa-miR-186-5p as a potential regulator of chondrocyte proliferation and inflammation, providing new insights into the pathogenesis of this condition. These findings deepen the understanding of the molecular mechanisms driving capsular contracture and offer new avenues for the clinical management of posttraumatic elbow stiffness.

#### 2.1.2 Formation of heterotopic ossification

Heterotopic ossification is the abnormal formation of mature lamellar bone within non-skeletal tissues. Clinically, it manifests through the differentiation of multipotent mesenchymal cells into osteoblasts, which subsequently produce osteoid, facilitate its mineralization, and drive osteogenesis (10). This process can mechanically impede joint mobility, and radiographic examination often reveals calcification of the heterotopic bone and increased density of the surrounding soft tissues. The Wnt/β-catenin signaling pathway is a well-established conserved pathway crucial for various fundamental processes. It plays a significant role in promoting angiogenesis during heterotopic ossification (HO) repair and influences the differentiation and maturation of osteoblasts and chondrocytes. Additionally, it crosstalks with BMP, Hedgehog, YAP, and other signaling pathways as well as miRNAs, collectively impacting HO development. Various compounds such as RARγ agonists, Wnt inhibitors, and Verteporfin have demonstrated the ability to hinder HO formation by modulating the Wnt/β-catenin signaling pathway. Nevertheless, there exists a research gap concerning the involvement of Wnt signaling pathways in the early inflammatory processes of HO; hence, solely manipulating the Wnt signaling pathway may not suffice to prevent HO (11–13).

### 2.2 Risk factors

#### 2.2.1 Original damage

The degree of elbow joint stiffness following trauma is influenced by the type of fracture and the initial injury energy. Celli et al. (14) investigated the impact of various elbow joint fractures, including supracondylar, intercondylar, intra-articular comminuted, condylar, capitellum humeral, olecranon, and radial capitellum fractures, on the anatomical structure of the joint, elucidating the underlying causes of elbow stiffness associated with different fracture types. Additionally, Zhang et al. (15) studied 169 patients with post-traumatic elbow stiffness and found that the initial injury energy level had a differential effect on the prognosis of elbow surgery. Specifically, high-energy injuries were identified as an independent risk factor for severe elbow stiffness, defined as a flexion and extension mobility range of more than 30 but less than or equal to 60°.

#### 2.2.2 Fixed time

After elbow trauma, although surgery can restore the original bony anatomy, repair the surrounding soft tissue. However, there is still controversy about the braking time. Studies have shown that when intra-articular fractures occur, the immobilization time exceeds 3 days, and the articular cartilage begins to be repaired by fibrous tissue; if the immobilization time exceeds 6–12 weeks, even if there is no damage, joint function will be significantly affected. At the same time, it was found that when the immobilization time after open reduction of elbow terror triad exceeded 2 weeks, the probability of elbow stiffness was 3.237 times (16). However, for conservative treatment, increasing the length of fixation may lead to stiffness, Monument et al. (17) suggested that prolonged plaster fixation is detrimental to elbow motion because it may stimulate capsular contracture and fibrosis and cause structural deformation in the periarticular region.

#### 2.2.3 Rehabilitation training

Rehabilitation following surgical treatment of elbow fractures is essential for the recovery of joint function. However, exceeding the patient's tolerance during rehabilitation training can damage the periarticular tissues, leading to inflammation, scarring, and restricted elbow mobility. Conversely, inappropriate training methods not only impede effective joint recovery but also exacerbate joint damage, resulting in uneven stress distribution and potential joint dislocation or subluxation. Given the heterogeneity in trauma severity and individual physical conditions, a one-size-fits-all rehabilitation approach is challenging and may even have adverse consequences. For severely injured elbows, a slower, more gradual recovery process may be warranted, while for patients with milder trauma, the rehabilitation timeline could be appropriately accelerated (18).

#### 2.2.4 Psycho-mental state

Psychological comorbidities can significantly impact the outcomes of surgical interventions for elbow dysfunction. A study of 108 patients reported that 40.7% exhibited mild to moderate depression, while 23. 1% experienced severe to severe depression. Additionally, 27.8% exhibited mild to moderate anxiety, and 25.9% exhibited severe to severe anxiety (19). Sun et al. (20) found that while patients generally achieved favorable short-term clinical outcomes following surgical release, those with preoperative Hospital Anxiety and Depression Scale (HADS) scores ≥11 demonstrated poorer outcomes compared to those with HADS scores <11. These findings underscore the importance of assessing and addressing psychological factors in the management of elbow dysfunction to optimize surgical results.

## 3 Classification and diagnosis

### 3.1 Classification

The Kay and Morrey classifications are commonly used to categorize post-traumatic elbow stiffness (Table 1). The Kay (21) classification is based on the presence, severity, and combination of the 3 most common factors causing stiffness (capsular contracture, articular fractures, heterotopic ossification) identifying the 5 clinical situations more frequent in clinical practice. The Morrey (22) classification divides post-traumatic elbow stiffness into intra-articular, extra-articular, and mixed types based on the anatomical location of the underlying pathology, among the subtypes, the mixed type exhibited the highest clinical frequency. The recently proposed S.T.I.F. classification system emphasizes the etiology of elbow stiffness and provides a diagnostic framework to better understand the natural history, guide surgical management, and predict clinical outcomes (23). However, there are few reports on the application of this classification method in clinical practice, and more practical applications are needed to confirm its effectiveness.

**Table 1:** Kay classification and Morrey classification.

Classification system	Type	Description/Components
Kay classification	Ⅰ	Simple soft tissue contracture
	ⅠⅠ	Soft tissue contracture combined with heterotopic ossification
	ⅠⅠⅠ	Non-displaced intra-articular fracture with soft tissue contracture
	ⅠV	Displaced intra-articular fracture with soft tissue contracture
	V	Post-traumatic Osteoarthritis
Morrey classification	Extrinsic factors	Capsular-ligament contracture, Muscle/tendon retraction, Ulnar nerve neuropathy, Heterotopic ossification, and Skin contracture
	Intrinsic factors	Intra-articular adhesions and Deformities, Cartilage lesions, Loose-bodies, and Impinging osteophytes
	Mixed	Both intrinsic and extrinsic elements

### 3.2 Diagnosis

**Figure 1:**
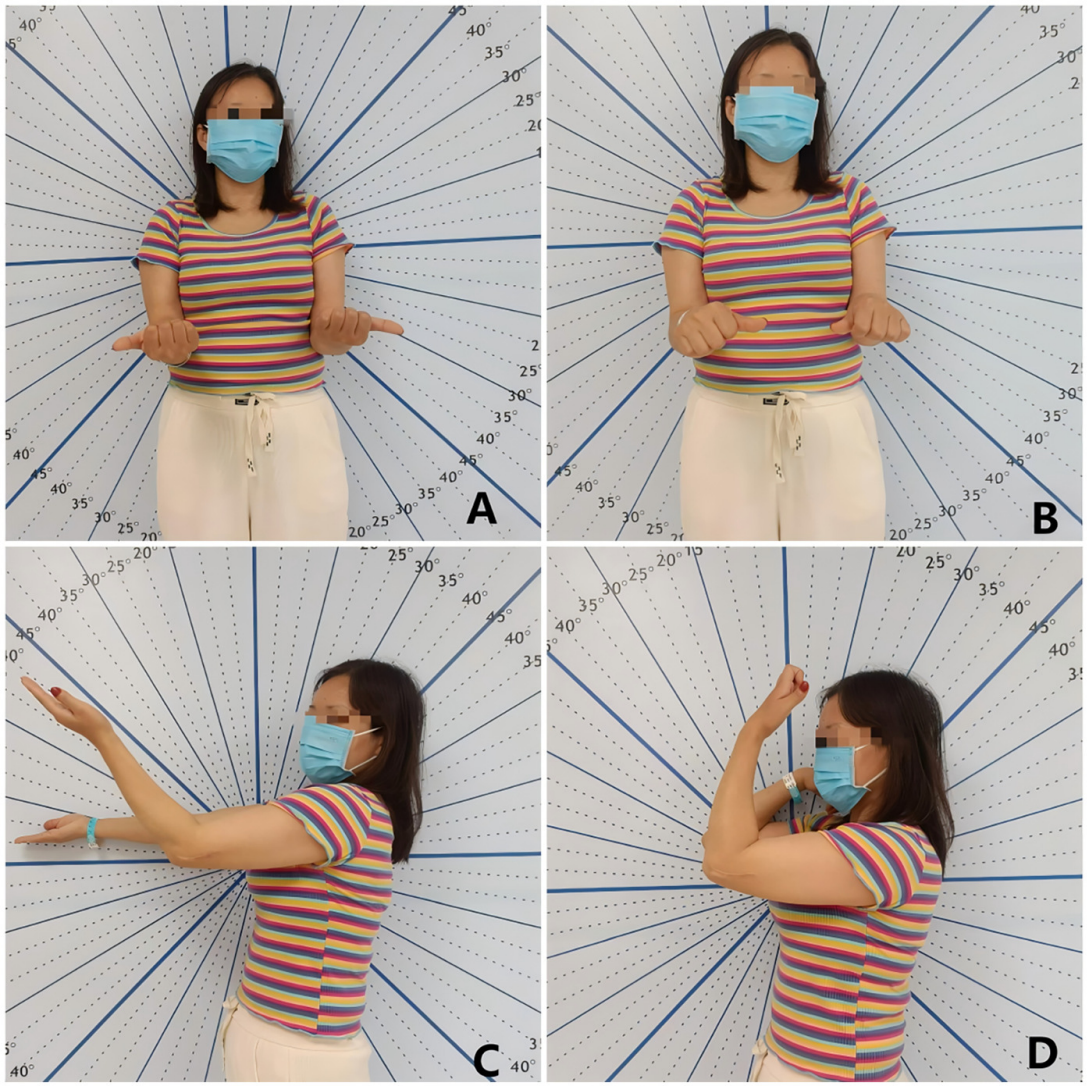
A 35-year-old female presented with elbow stiffness 16 months after surgery for a distal humerus fracture. **(A,B)** Preoperative assessment of elbow rotational function showed 80° of pronation and 90° of supination. **(C,D)** Preoperative assessment of elbow extension and flexion function revealed 45° of extension and 95° of flexion.

**Figure 2:**
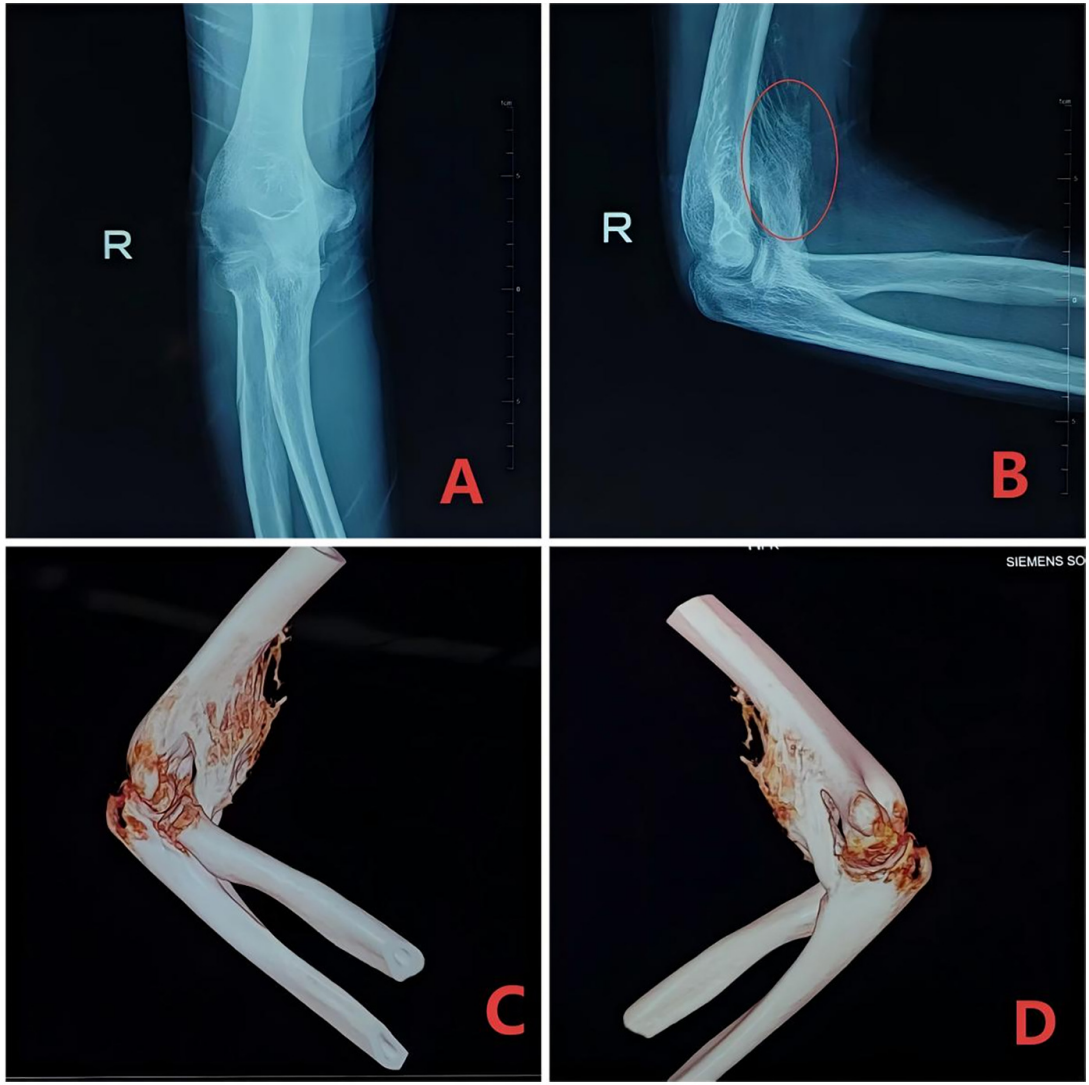
A 24-year-old male presented with right elbow stiffness for 4 months. Heterotopic ossification was observed adjacent to the right ulnar coronoid process. The articular relationships were approximately normal, with no significant widening or narrowing of the joint space. **(A,B)** Anteroposterior and lateral x-ray films. **(C,D)** Three-dimensional imaging of the right elbow joint.

The comprehensive assessment of the patient's condition is essential for effective treatment and rehabilitation. This should involve a detailed investigation of the original traumatic event, the treatment process, and the rehabilitation program. Additionally, an in-depth understanding of the patient's daily activities and upper limb functional requirements is crucial (Figure 1). The physical examination should focus on the appearance of the elbow joint, including any swelling, deformity, or muscle atrophy, as well as the range of motion and pain levels. Importantly, the assessment should also consider the presence of any neurological symptoms, as these may contribute to increased pain and limb dysfunction, ultimately impacting the patient's recovery and quality of life. The Mayo Elbow Performance Score (MEPS) is a widely adopted tool for assessing elbow function in patients with post-traumatic elbow stiffness. It evaluates four key domains: pain, range of motion, stability, and the ability to perform activities of daily living. The total score ranges from 0 to 100, with higher scores indicating better elbow function. In contrast, the Shanghai Elbow Dysfunction Score (SHEDS) provides a more comprehensive evaluation of elbow joint function. It assesses three aspects: elbow joint mobility, elbow-related symptoms, and patient satisfaction, including the patient's subjective perceptions. This multidimensional approach is crucial for thoroughly evaluating the treatment outcomes and quality of life in these patients (24). The anterolateral radiographic evaluation is essential for assessing the bony architecture of the elbow joint, including fracture healing, malunion, nonunion, and joint space changes. Computed tomography (CT) imaging, however, surpasses radiography in identifying and characterizing the osseous causes of elbow stiffness. CT can accurately depict the location and morphology of heterotopic ossification (HO) and osteophytes with a sensitivity of 92%, and it can also precisely delineate the joint space, which aids in the diagnosis of advanced osteoarthritis (OA) (25) (Figure 2).

## 4 Treatments

### 4.1 Nonoperative treatment

#### 4.1.1 Rehabilitation training

The primary objective of rehabilitation following elbow joint trauma is to restore a functional, pain-free, and stable elbow joint for patients. This entails comprehensive training regimens encompassing active and passive range-of-motion exercises, as well as muscle strengthening protocols. Rehabilitation is a protracted process, typically spanning at least 6 months, and its success is contingent upon the patient's active engagement and cooperation with the healthcare provider (26).

#### 4.1.2 Physical therapy

Hyperthermia, ultrasound therapy, and extracorporeal shock wave therapy are effective physical therapy modalities for the management of post-traumatic elbow stiffness. Hyperthermia can relieve muscle spasms, alleviate pain, soften scar tissue, and create favorable conditions for rehabilitation training. Ultrasound therapy, through its mechanical, thermal, and cavitation effects, can promote local blood circulation, reduce inflammation, and soften scar tissue, thereby improving elbow joint mobility. Chen et al. (27) demonstrated the effectiveness and safety of extracorporeal shock wave therapy combined with bracing in the treatment of post-traumatic elbow stiffness through a case-control study, providing robust evidence for the clinical application of this approach. Furthermore, the combination of shock wave therapy and surface electromyographic biofeedback training has been shown to effectively relieve pain, improve elbow function, and enhance active joint mobility in patients with post-traumatic elbow stiffness (28).

#### 4.1.3 Graded motor imagery

Graded motor imagery (GMI) is a therapeutic intervention designed to enhance functional recovery without exacerbating pain. The approach involves a series of graded motor cognitive strategies, including left-right discrimination (implicit motor imagery), imaginary movement (explicit motor imagery), and mirror therapy. By minimizing the detrimental effects of pain-related exercise phobias, GMI can help patients regain pre-injury functional levels. A randomized controlled trial by Birinci et al. (29) demonstrated that incorporating GMI into a rehabilitation program following elbow fracture surgery significantly reduced pain intensity, pain-related exercise fear, and improved functional outcomes in patients with post-traumatic elbow stiffness.

#### 4.1.4 Traditional Chinese medicine treatment

The extant literature suggests that traditional Chinese medicine (TCM) interventions, including internal and external administration of herbal remedies, fumigation, manipulation, acupuncture, and massage, exhibit efficacy in restoring elbow joint function in patients with post-traumatic elbow stiffness. These TCM modalities have been shown to improve range of motion and overall elbow joint mobility (2). Furthermore, integrating TCM therapies with other physical and surgical treatments may yield enhanced outcomes for this patient population.

### 4.2 Operative treatment

#### 4.2.1 Indications of operation

**Figure 3:**
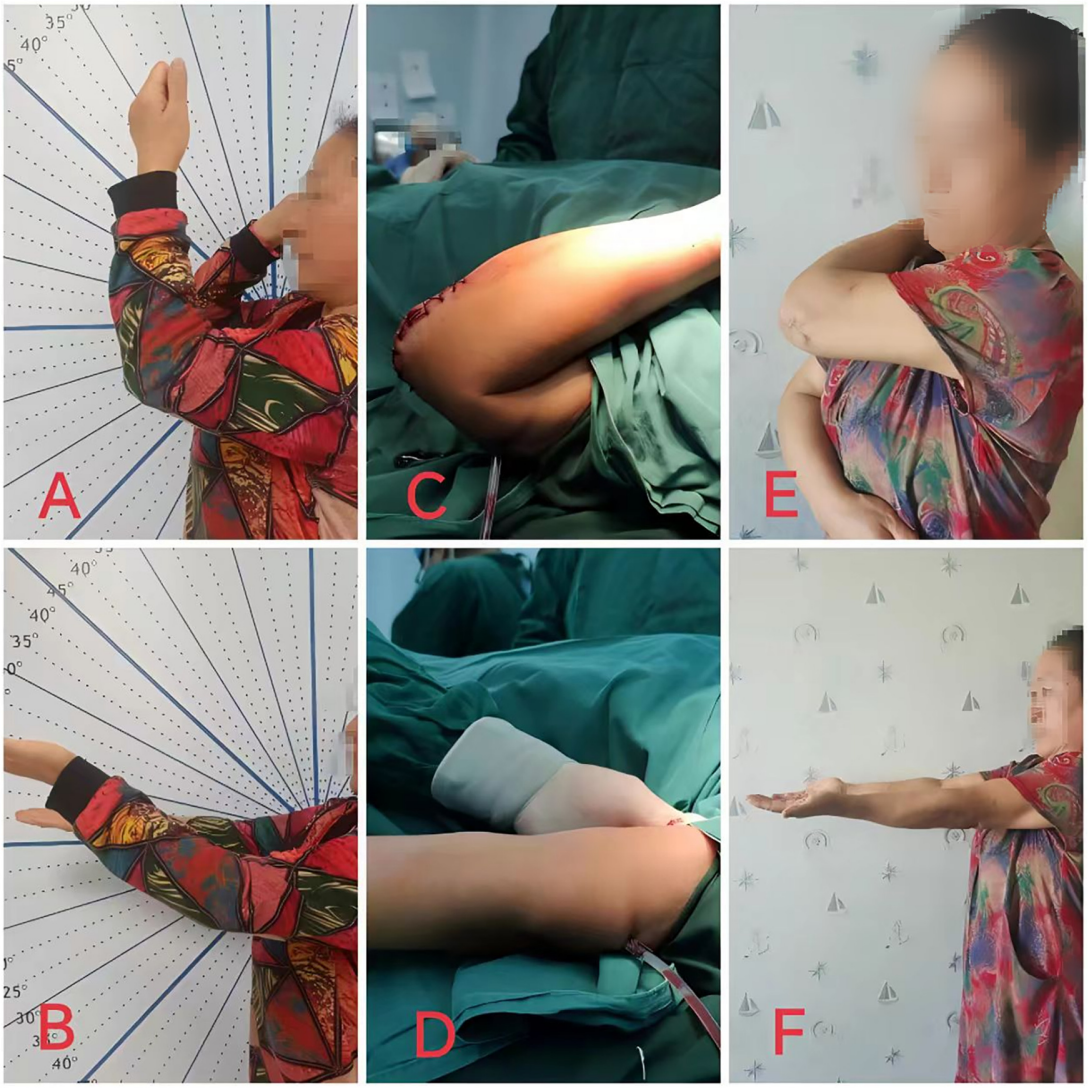
A 56-year-old female patient with post-traumatic elbow stiffness for 12 months underwent open release surgery through the original posterior surgical scar incision of the left elbow. **(A,B)** Photographs demonstrating the flexion and extension function of the left elbow joint before surgery; **(C,D)** photographs showing the function of the left elbow joint during surgery; **(E,F)** photographs illustrating the flexion and extension function of the elbow joint at 3 months after surgery.

Patients with post-traumatic elbow stiffness characterized by an extension limitation greater than 30° and a flexion angle less than 130° exhibit a markedly restricted range of motion, which can significantly impair daily activities and occupational performance. For those who fail to achieve substantial improvement after 6 months of conservative management, surgical release may be warranted (6) (Figure 3). However, for individuals with high functional demands on the elbow joint, such as athletes or those in specialized occupations, the criteria for surgical intervention may be relaxed. Even if conservative treatment yields functional improvements, surgical treatment can be considered if the restored range of motion remains insufficient to meet the patient's specific needs.

#### 4.2.2 Operation opportunity

The optimal timing for surgical intervention to address post-traumatic elbow stiffness remains a subject of debate. While some clinicians advocate delaying surgery for 6–8 months or even up to 1 year after the initial trauma, this approach is primarily driven by concerns regarding the potential for increased surgical risk and complications associated with early intervention. During the initial post-traumatic period, the affected elbow joint may exhibit localized inflammation, swelling, and instability, which could potentially increase the complexity of the surgical procedure and the likelihood of adverse outcomes (30). Conversely, several studies have suggested that early surgical intervention, performed within 6–10 months of the initial injury, may not necessarily result in poorer outcomes compared to intermediate (11–20 months) or late (>20 months) surgical timing. These investigations have found no significant differences in postoperative elbow mobility, functional scores, or complication rates among the different surgical timing groups. Importantly, early surgery has been associated with shorter recovery periods and earlier return to work, potentially offering advantages for certain patients. Therefore, while the optimal timing for surgical management of post-traumatic elbow stiffness remains a subject of ongoing debate, the available evidence suggests that early intervention, performed within the first 6–10 months after the initial injury, may be a viable and potentially beneficial approach in selected cases, provided that the local inflammatory and stability conditions are appropriately addressed (31).

#### 4.2.3 Surgical methods

##### 4.2.3.1 Open arthrolysis

The medial approach enables targeted management of elbow joint pathologies. This surgical technique facilitates careful dissection and decompression of the ulnar nerve, while concurrently allowing release of the medial joint capsule, ligaments, and other involved structures. This approach is well-suited for addressing stiffness attributed to abnormal medial elbow anatomy, such as medial collateral ligament contracture, ulnar nerve compression, and other pathological changes.

The lateral approach to the elbow joint typically involves an incision near the lateral epicondyle of the humerus, which allows for improved exposure of the lateral joint structures, including the lateral collateral ligament and radial head. This surgical approach enables the release of the lateral joint capsule and ligaments, as well as the potential for radial head resection or the management of heterotopic ossification, if required. This approach is well-suited for addressing stiffness resulting from abnormalities in the lateral joint structures, such as contractures of the lateral collateral ligament or malunion of radial head fractures. The lateral approach provides excellent visualization of the lateral and anterior articular surfaces, but offers limited exposure to medial and posterior lesions. A previous study (32) has reported the use of a modified lateral approach, which involves dissection between the extensor carpi radialis brevis and extensor carpi radialis longus tendons, with good outcomes. This modified technique allows for improved visualization of the radial nerve and facilitates the release and synovectomy of the elbow joint.

The combined medial and lateral approach leverages the benefits of both the medial and lateral approaches to enable more comprehensive exposure of the elbow's anatomical structures. During the surgical procedure, the medial and lateral joint capsules, ligaments, and muscles can be released concurrently. If necessary, ulnar nerve advancement and radial head resection can also be performed. This approach is well-suited for the management of complex post-traumatic elbow stiffness, particularly when both medial and lateral structural abnormalities are present. Existing evidence indicates that the combined medial and lateral approach for surgical release of post-traumatic elbow stiffness can yield satisfactory outcomes, increasing elbow flexion and extension range of motion, as well as improving forearm rotation range of motion in patients with elbow stiffness and limited forearm rotation following radial head fracture internal fixation (33).

The administration of tranexamic acid during elbow arthrolysis procedures has been demonstrated to effectively mitigate postoperative bleeding or drainage without concomitantly elevating the risk of thrombotic complications (34). This, in turn, facilitates expedited patient recovery and enhances postoperative satisfaction. Furthermore, the combined approach of botulinum toxin injection and open lysis has been shown to significantly improve clinical outcomes, particularly in the management of traumatic elbow stiffness secondary to heterotopic ossification (35).

##### 4.2.3.2 Arthroscopic arthrolysis

Arthroscopic lysis, a minimally invasive surgical approach, offers several advantages over traditional open surgery, including smaller incisions, reduced tissue trauma, and faster postoperative recovery (36). The enhanced visualization provided by arthroscopy enables precise identification and treatment of diseased joint tissues. Patients can also commence rehabilitation training earlier to restore elbow range of motion and function. However, arthroscopic treatment may be limited in cases of severe joint deformities, extensive heterotopic ossification, or extreme elbow stiffness, which may require a combined open surgical approach (37). When addressing elbow joint stiffness arthroscopically, safety should be the primary concern, with thorough intraoperative cleaning and release, and careful control of the duration of anterior and posterior compartment procedures (38). The indications for arthroscopic surgery are relatively constrained, as the surgical outcomes can be influenced by the underlying etiology, fracture location, patient age, and disease course (39). Additionally, the technique demands substantial surgeon experience and technical proficiency, necessitating further research and development to refine the procedure.

## 5 Early post-operative rehabilitation management

Postoperative rehabilitation management following elbow surgery aims to maintain the maximum range of motion (ROM) achieved during the procedure and maximize joint movement and muscle strength. Early initiation of rehabilitation exercises, typically within 48 h after surgery, is widely advocated as particularly important. Existing evidence suggests that the recovery of ROM after surgical release of posttraumatic elbow stiffness is not dependent on the pre-operative, intraoperative, or 2-week postoperative ROM, but rather, the majority of ROM recovery occurs in the early postoperative period, with the maximum ROM expected to be reached around 16 weeks after the surgical intervention (40).

Pain is a source of anxiety after traumatic elbow stiffness release, and pain-related anxiety can further increase perceived pain intensity and lead to pain-related fear of movement. Furthermore, fear of motion-related pain often leads to an unwillingness to perform required rehabilitation tasks, which may lead to behavioral avoidance of movement and activities, and according to the fear of safety perspective, attention to pain control in the initial stages of rehabilitation is critical (41). Some scholars have shown that a multimodal analgesia scheme using ropivacaine brachial plexus continuous block and sufentanil self-controlled analgesia after traumatic elbow joint release can improve patient comfort and rehabilitation compliance (42). In addition, the use of nonsteroidal anti-inflammatory drugs and indomethacin can prevent heterotopic ossification, relieve pain and inflammation, and promote elbow function recovery. Combined with low-dose radiation therapy, it can better prevent postoperative recurrence of heterotopic ossification (43).

Postoperative treatment regimens incorporating various orthotic devices can enhance functional recovery of the elbow joint. Scholars have utilized tension bracing in conjunction with open elbow release to manage elbow joint stiffness. At final follow-up, patients treated with this approach demonstrated significantly greater elbow flexion and extension range of motion as well as improved Mayo Elbow Performance Scores compared to controls, indicating that tension bracing combined with open release can effectively improve elbow mobility and function with satisfactory outcomes (44). Similarly, Wei et al. (45) found that patients treated with open lysis and a customized 3D-printed hinged brace, followed by manual rehabilitation, exhibited superior active elbow flexion-extension and Mayo scores relative to a control group after surgery, with statistically significant differences.

## 6 Conclusions

In conclusion, advancements have been made in the diagnosis and treatment of posttraumatic elbow stiffness. However, further research is necessary to explore additional avenues for both research needs and clinical practice.

In research needs, while interventional studies on the Wnt/β-catenin signaling pathway have shown promise in animal models, clinical trials investigating TGF-β inhibitors for elbow contracture are limited. Current research predominantly focuses on postoperative adjuvant therapies, such as the combination of nonsteroidal anti-inflammatory drugs and radiotherapy to reduce heterotopic ossification and TGF-β expression. Future investigations should delve into more precise targeted interventions, including subtype-specific inhibitors, and the development of individualized treatment protocols. Additionally, newly identified hinge genes associated with joint capsule contraction (SPP1, IBSP, MMP13, MYO1A), crucial microRNAs (has-miR-186-5p, has-miR-515-5p, hsa-miR-590-3p), and transcription factors (TFDP1, STAT3) hold potential as prognostic and therapeutic targets, offering novel research avenues. Nevertheless, the role of psychological factors in these mechanisms remains unclear, warranting further research for perioperative intervention.

In clinical applications, the newly proposed S.T.I.F classification system requires further validation of its clinical utility through additional clinical trials. Leveraging advancements in Internet and AI technologies, real-time patient activity data can be collected via Internet-connected devices. This data can be utilized in conjunction with AI algorithms to tailor personalized rehabilitation programs, ultimately aiming to mitigate postoperative readmission rates. Future research endeavors will emphasize interdisciplinary collaboration among healthcare professionals to engage in comprehensive patient evaluation, treatment planning, and rehabilitation strategizing. This approach aims to establish a treatment framework that integrates orthopedics, rehabilitation, and psychology, facilitating a personalized, systematic, and standardized diagnostic and therapeutic process. Such efforts are geared towards enhancing patients' quality of life, expediting their return to normal daily activities and employment.

## References

[1] Liu SY Liu G. How to prevent stiffness after elbow joint injury. J Trauma Surg. (2023) 25(8):639–40. 10.3969/j.issn.1009-4237.2023.08.014

[2] Ma CY Zhai Y Hu L. Research progress in treatment of post-traumatic elbow stiffness. J Clin Orthop Res. (2021) 6(4):250–2. 10.19548/j.2096-269x.2021.04.014

[3] Zhang D Nazarian A Rodriguez EK. Post-traumatic elbow stiffness: pathogenesis and current treatments. Shoulder Elbow. (2020) 12(1):38–45. 10.1177/1758573218793903 32010232 PMC6974890

[4] Siemensma MF van der Windt AE van Es EM Colaris JW Eygendaal D. Management of the stiff elbow: a literature review. EFORT Open Rev. (2023) 8(5):351–60. 10.1530/EOR-23-0039 37158372 PMC10233805

[5] Editorial Committee of Chinese Journal of Hand Surgery. Expert consensus on diagnosis and treatment of adult traumatic elbow stiffness release. Chin J Hand Surg. (2020) 36(1):3–10. 10.3760/cma.j.issn.1005-054X.2020.01.002

[6] Cohen MS Schimmel DR Masuda K Hastings H 2nd Muehleman C. Structural and biochemical evaluation of the elbow capsule after trauma. J Shoulder Elbow Surg. (2007) 16(4):484–90. 10.1016/j.jse.2006.06.018 17368926 PMC2080784

[7] Lee DR Therrien E Song BM Camp CL Krych AJ Stuart MJ Arthrofibrosis nightmares: prevention and management strategies. Sports Med Arthrosc Rev. (2022) 30(1):29–41. 10.1097/JSA.0000000000000324 35113841 PMC8830598

[8] Xiao D Liang T Zhuang Z He R Ren J Jiang S Lumican promotes joint fibrosis through TGF-β signaling. FEBS Open Bio. (2020) 10(11):2478–88. 10.1002/2211-5463.12974 32910552 PMC7609791

[9] Liu N Dong J Li L Xu J Yang C Yu Z Novel clinical insights into the pathogenesis of posttraumatic elbow stiffness: an expression profile analysis of contracted joint capsule in human. J Inflamm Res. (2025) 18:167–82. 10.2147/JIR.S499986 39802512 PMC11721169

[10] Jayamaraju D Sarkar AS Patra SK Palanivelayutham SK Rajasekaran S. A surgical protocol for management of post traumatic heterotopic ossification of elbow. Indian J Orthop. (2021) 55(4):898–906. 10.1007/s43465-021-00381-x 34194645 PMC8192647

[11] Bei M Cao Q Zhao C Xiao Y Chen Y Xiao H Heterotopic ossification: current developments and emerging potential therapies. Chin Med J (Engl). (2025) 138(4):389–404. 10.1097/CM9.0000000000003244 39819765 PMC11845195

[12] Li SN Ran RY Chen J Liu MC Dang YM Lin H. Angiogenesis in heterotopic ossification: from mechanisms to clinical significance. Life Sci. (2024) 351:122779. 10.1016/j.lfs.2024.122779 38851421

[13] Zhao Y Liu F Pei Y Lian F Lin H. Involvement of the wnt/β-catenin signalling pathway in heterotopic ossification and ossification-related diseases. J Cell Mol Med. (2024) 28(18):e70113. 10.1111/jcmm.70113 39320014 PMC11423343

[14] Celli A Prandini M Cheli A Pederzini LA. Elbow stiffness: arthritis and heterotopic ossification. J ISAKOS. (2024) 9(1):103–14. 10.1016/j.jisako.2023.10.009 37879605

[15] Zheng W Liu J Song J Fan C. Risk factors for development of severe post-traumatic elbow stiffness. Int Orthop. (2018) 42(3):595–600. 10.1007/s00264-017-3657-1 28988397

[16] He X Fen Q Yang J Lei Y Heng L Zhang K. Risk factors of elbow stiffness after open reduction and internal fixation of the terrible triad of the elbow joint. Orthop Surg. (2021) 13(2):530–6. 10.1111/os.12879 33619861 PMC7957406

[17] Qian Y Yu S Shi Y Huang H Fan C. Risk factors for the occurrence and progression of posttraumatic elbow stiffness: a case-control study of 688 cases. Front Med (Lausanne). (2020) 7:604056. 10.3389/fmed.2020.604056 33392226 PMC7772462

[18] Zhao H. Effect of individualized rehabilitation training on elbow function recovery after distal humerus fracture. China Health Care Nutr. (2020) 30(7):146.

[19] Liu W Sun Z Xiong H Liu J Lu J Cai B What are the prevalence of and factors independently associated with depression and anxiety among patients with posttraumatic elbow stiffness? A cross-sectional, multicenter study. J Shoulder Elbow Surg. (2022) 31(3):469–80. 10.1016/j.jse.2021.11.014 34968692

[20] Sun W Chen C Jiang X Hua K Zha Y Gong M Anxiety and depression are associated with poor outcomes in open elbow arthrolysis. Injury. (2023) 54(8):110713. 10.1016/j.injury.2023.03.041 37270347

[21] Mellema JJ Lindenhovius AL Jupiter JB. The posttraumatic stiff elbow: an update. Curr Rev Musculoskelet Med. (2016) 9(2):190–8. 10.1007/s12178-016-9336-9 26984466 PMC4896879

[22] Celli A Pederzini LA Morrey BF. Elbow stiffness: interview with professor Bernard Morrey. J ISAKOS. (2024) 9(1):94–7. 10.1016/j.jisako.2023.09.002 37696358

[23] Marinelli A Guerra E Bain G. Classification of elbow stiffness. J ISAKOS. (2024) 9(2):234–9. 10.1016/j.jisako.2023.10.011 37923144

[24] Birinci T Altun S Ziroğlu N Kaya Mutlu E. The Shanghai elbow dysfunction score: psychometric properties, reliability and validity study of the Turkish version. Eval Health Prof. (2024) 47(1):111–8. 10.1177/01632787231183089 37312232

[25] Lombard C Teixeira P Germain E Dodin G Louis M Blum A Elbow stiffness imaging: a practical diagnostic and pretherapeutic approach. J Clin Med. (2021) 10(22):5348. 10.3390/jcm10225348 34830630 PMC8622234

[26] Jones V. Conservative management of the post-traumatic stiff elbow: a physiotherapist’s perspective. Shoulder Elbow. (2016) 8(2):134–41. 10.1177/1758573216633065 27583012 PMC4950468

[27] Chen W. Observation on the therapeutic effect of extracorporeal shock wave combined with static joint stretching training on elbow joint stiffness aftertrauma. China Modern Med. (2024) 31(27):80–4. 10.3969/j.issn.1674-4721.2024.27.019

[28] Fang LY Ren YC Cao YH Wang HW. Effect of extracorporeal shock wave therapy combined with surface EMG biofeedback on post-traumatic stiffness of the elbow. J Pract Med. (2024) 40(10):1364–9. 10.3969/j.issn.1006-5725.2024.10.006

[29] Birinci T Mutlu EK Altun S. The efficacy of graded motor imagery in post-traumatic stiffness of elbow: a randomized controlled trial. J Shoulder Elbow Surg. (2022) 31(10):2147–56. 10.1016/j.jse.2022.05.031 35803550

[30] Lanzerath F Wegmann K Hackl M Uschok S Ott N Müller LP Surgical arthrolysis of the stiff elbow: a systematic review. Arch Orthop Trauma Surg. (2023) 143(5):2383–93. 10.1007/s00402-022-04442-0 35482109 PMC10110632

[31] Sun Z Cui H Liang J Li J Wang X Fan C. Determining the effective timing of an open arthrolysis for post-traumatic elbow stiffness: a retrospective cohort study. BMC Musculoskelet Disord. (2019) 20(1):122. 10.1186/s12891-019-2506-3 30909899 PMC6434886

[32] Roulet S Charruau B Mazaleyrat M Ferembach B Marteau E Laulan J Modified lateral approach of the elbow for surgical release and synovectomy. Tech Hand Up Extrem Surg. (2020) 25(2):84–8. 10.1097/BTH.0000000000000312 32868694

[33] Guo YB Zhang XL Zheng YH Chen ZM Chen KY Ye CX. Combined medial and lateral approach for surgical release in 23 cases of traumatic elbow stiffness. Chin J Bone Joint Inj. (2023) 38(7):758–60. 10.7531/j.issn.1672-9935.2023.07.023

[34] Zhang B Zhang W Xu J Ding J. Effect of topical tranexamic acid on post-traumatic elbow stiffness in patients treated with open arthrolysis: a prospective comparative study. J Shoulder Elbow Surg. (2020) 29(7):1375–9. 10.1016/j.jse.2020.02.010 32418856

[35] Freibott CE Bäcker HC Shoap SC Tedesco LJ Galle SE Rosenwasser MP. Treatment methods for post-traumatic elbow stiffness caused by heterotopic ossification. J Shoulder Elbow Surg. (2020) 29(7):1380–6. 10.1016/j.jse.2020.02.026 32553438

[36] Dai J Zhang G Li S Xu J Lu J. Arthroscopic treatment of posttraumatic elbow stiffness due to soft tissue problems. Orthop Surg. (2020) 12(5):1464–70. 10.1111/os.12787 33015918 PMC7670133

[37] Yang JS Xiang M Zhang Q Dai F. Clinical effect of arthroscopic combined with open release in the treatment of severe and extremely severe post-traumatic elbow stiffness. Chin J Orthop. (2022) 42(4):236–43. 10.3760/cma.j.cn121113-20211020-00610

[38] Ghayyad K Ahmadi Z Rajabi H Alimohammadi MM Kachooei AR. Arthroscopic capsular release for post-traumatic elbow stiffness. Cureus. (2023) 15(10):e47838. 10.7759/cureus.47838 38021529 PMC10676772

[39] Meng CQ Wang H Zhang QM Jin SY Huang W. Analysis of influencing factors of total arthroscopic release for elbow stiffness. Acta Med Univ Sci Technol Huazhong. (2019) 48(4):450–3. 10.3870/j.issn.1672-0741.2019.04.015

[40] Ahmad F Torres-Gonzales L Mehta N Cohen MS Simcock X Wysocki RW. Progression patterns of range of motion progression after open release for post-traumatic elbow stiffness. JSES Int. (2022) 6(3):545–9. 10.1016/j.jseint.2022.02.005 35572429 PMC9091921

[41] Osumi M Sumitani M Nishi Y Nobusako S Dilek B Morioka S. Fear of movement-related pain disturbs cortical preparatory activity after becoming aware of motor intention. Behav Brain Res. (2021) 411:113379. 10.1016/j.bbr.2021.113379 34051229

[42] Deng JL Wu HS Zhang YH Wu X Cai CF Xu JK Effect analysis of multimodal analgesia in rehabilitation after release of traumatic elbow stiffness. Chin J Bone Joint Inj. (2019) 34(3):314–6. 10.7531/j.issn.1672-9935.2019.03.036

[43] Atwan Y Abdulla I Grewal R Faber KJ King GJW Athwal GS. Indomethacin for heterotopic ossification prophylaxis following surgical treatment of elbow trauma: a randomized controlled trial. J Shoulder Elbow Surg. (2023) 32(6):1242–8. 10.1016/j.jse.2023.02.119 36907317

[44] Xiong C Zhang K He X Yang JR He CJ Huang W Preliminary efficacy analysis of tension brace combined with open arthrolysis in the treatment of elbow ankylosis. Chin J Shoulder Elbow (ElectronEd). (2021) 9(3):257–62. 10.3877/cma.j.issn.2095-5790.2021.03.011

[45] Wei SY Ma JZ Ma N Wang C Xue LY Luo XH Study on the application effect of personalized 3D printed hinge brace after elbow release. Ningxia Med J. (2024) 46(9):799–801. 10.13621/j.1001-5949.2024.09.0799

